# Influence of Resistance Exercise on Appetite and Affect Following Pre-Sleep Feeding

**DOI:** 10.3390/sports6040172

**Published:** 2018-12-11

**Authors:** Takudzwa A. Madzima, Jonas R. Black, Jared T. Melanson, Svetlana Nepocatych, Eric E. Hall

**Affiliations:** Department of Exercise Science, Energy Metabolism and Body Composition Laboratory, Elon University, 100 Campus Drive, Elon, NC 27244, USA; jblack14@elon.edu (J.R.B.); jmelanson@elon.edu (J.T.M.); snepocatch@elon.edu (S.N.); ehall@elon.edu (E.E.H.)

**Keywords:** affect, cortisol, appetite, resistance exercise, pre-sleep feeding

## Abstract

To determine changes in appetite, affect and cortisol in response to an acute bout of resistance exercise (RE) the morning after consuming whey (WP) and casein (CP) protein and a non-caloric placebo (PLA) consumed pre-sleep, 14 active men (n = 5) and women (n = 9) consumed a single dose of 24 g WP, 48 g WP, 24 g CP, 48 g CP, or PLA 30 min pre-sleep. Prior to and immediately after RE, appetite, affect and cortisol were assessed. Significant time effects were observed for Energetic Arousal and Tense Arousal (*p* = 0.017) and Feeling Scale and Felt Arousal Scale (*p* < 0.001). Appetite did not change over time or condition. Cortisol levels increased after RE (*p* = 0.007). Pre-RE, Tense Arousal was correlated with hunger (r = 0.25, *p* = 0.047) and desire to eat (r = 0.35, *p* = 0.005). Post-RE, cortisol was found to be significantly related to Feeling Scale (r = 0.32, *p* = 0.018), Felt Arousal Scale (r = 0.33, *p* = 0.015) and Energetic Arousal (r = 0.32, *p* = 0.018). Varying doses of WP and CP pre-sleep did not have an effect on morning appetite and cortisol, but cortisol was found to be related to affect and appetite.

## 1. Introduction

Recently, consumption of protein during the hours in close proximity to sleep has been considered a new feeding strategy in nutrient timing research [1,2]. Protein’s ability to increase satiety, a component of appetite, to a greater degree than carbohydrates and fats during the daytime is well documented [3,4]. The milk proteins casein (CP) and whey (WP) have been commonly consumed by active individuals, with CP often chosen as the ideal pre-sleep protein source due to the slower digestion and absorption rates when compared to WP [5,6]. This results in a more sustained rise in blood amino acid concentrations [5] that may be more optimal for the prolonged overnight period, thereby increasing overnight muscle protein synthesis and ultimately promoting recovery [7,8]. In contrast, WP elicits a more rapid increase in plasma amino acid concentrations, thus WP has been often recommended to be consumed in close proximity to resistance exercise [9,10]. However, the role of CP and WP to modulate appetite and affective mood states is still unclear. 

Although CP during daytime consumption has been shown to increase satiety and reduce hunger to a greater extent than WP [4,11], not all studies have observed this difference [12]. Recent work by Madzima et al. [13] did not observe a difference between isocaloric 30 g of CP or WP consumed pre-sleep on appetite the next morning in physically active males. Similarly, no differences in appetite were observed in obese women after consuming 30 g of CP or WP pre-sleep [14]. However, it is yet to be determined whether pre-sleep CP and WP exert similar effects on next-morning appetite before and after an exercise bout. Further, it is unclear whether any differences in appetite responses exist when CP and WP are consumed at a dose greater than the previously used 30 g [13,14]. To date, only one study has investigated the effect of pre-sleep feeding on next-morning appetite prior to a 10-km treadmill time trial [15]. They found that a pre-sleep low-calorie (~180 kcals) protein source (12 g) in the form of chocolate milk did not alter next morning appetite compared to a non-caloric placebo.

Previous studies have found appetite and affective states to be related to one another [16,17]. This may be important because affective responses to exercise have been hypothesized to be a predictor of adherence to exercise [18]. Acute bouts of aerobic exercise have consistently shown affect and mood to become more positive [19,20,21]. Similarly, acute bouts of resistance exercise have been found to lead to more positive affect [22,23]; however, the results are less consistent compared to aerobic exercise [24]. 

To the best of our knowledge no studies have examined the effect of pre-sleep eating on the affective responses before and after resistance exercise (RE). In addition, less is known in regards to the optimal pre-sleep dose of either CP or WP that can impact next-morning affect or the secretion of the catabolic stress hormone cortisol. Cortisol acts through various catabolic mechanisms, including the stimulation of protein breakdown, lipolysis and gluconeogenesis, thereby releasing amino acids and glucose into circulation to maintain blood glucose and thus explaining the reason it peaks in the morning [25,26]. Exercise, often described as a stressor on the body, can potentially influence appetite as stress often manifesting in the secretion of cortisol can both increase and decrease appetite [27]. Cortisol has also been found to influence affect, with some studies showing a relationship with negative affect [28,29,30] and positive affect [30,31,32,33]. A study by Rudolph and McAuley [34] found a negative relationship between positive affect and cortisol following aerobic exercise. Stimulation of the hypothalamic–pituitary–adrenal axis during exercise increases levels of cortisol [35], and the number of sets, volume, intensity and rest intervals during resistance exercise may have an effect on acute cortisol responses [36,37]. Previously, it has been shown that protein and carbohydrate supplementation may attenuate the cortisol response to exercise, however, it is questionable whether this was due to supplementation or altered hypothalamic function in response to heavy training [38]. Therefore, it is plausible that the provision of pre-sleep protein may reduce the need for protein breakdown to release amino acids for gluconeogenesis, thereby attenuating the rise of cortisol when performing resistance exercise in a fasted state the following morning. Considering many active adults perform exercise in a fasted state in the morning, it is likely that the elevations in circulating levels of cortisol during periods of low energy availability (such as prior to breakfast) may be attenuated by pre-sleep nutrition.

Therefore, the purpose of the present study is to investigate the effect of consuming 24 g and 48 g of CP and WP and a non-caloric placebo (PLA) pre-sleep on next-morning appetite, affect and cortisol before and after a RE bout. Due to the findings of some daytime studies demonstrating the superior satiating effect of CP [4,11] and its slow digesting nature [5], we hypothesize that 48 g CP, at a dose greater than the previously investigated 30 g, will result in a sustained release of amino acids overnight that will lead to greater feelings of satiety and positive affect before and in response to RE the next morning when compared to WP and PLA. We hypothesize that pre-sleep consumption of protein (CP or WP) would be superior to PLA (which will simulate not eating anything pre-sleep) and help mitigate the negative affective responses to exercise in a fasted state.

## 2. Materials and Methods

### 2.1. Participants

Fourteen physically active men (n = 5; age: 21.6 ± 2.0 years; body fat: 17.7% ± 5.7%) and women (n = 9; age: 25.3 ± 6.4 years; body fat: 22.0% ± 6.2%) participated in this study (Table 1). Participants were required to have regularly resistance trained for a minimum of two days per week for at least one year to be eligible to participate. Participants were excluded if they had: (1) uncontrolled hypertension (blood pressure (BP) > 160/100 mmHg) or taking BP medications; (2) had been diagnosed with cardiovascular disease, stroke, diabetes, thyroid, or kidney dysfunction; (3) or had dairy allergies. Additionally, current smokers or individuals taking cholesterol medication were excluded from participating. Eligible participants were asked to maintain their regular resistance and aerobic exercise regimens for the duration of the study. Participants that were consuming any nutritional supplements (except for a multivitamin) were asked to refrain from taking the supplements at least two weeks prior to their first visit and for duration of the study. The present study was conducted according to the Declaration of Helsinki guidelines, and was approved by the Elon University Institutional Review Board. Written informed consent was obtained before participation in the study. 

### 2.2. Study Design

**Figure 1:**
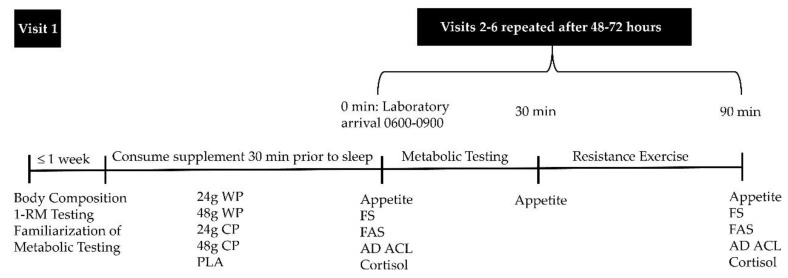
Study Timeline. 1-RM, one-repetition strength testing; WP, whey protein; CP, casein protein; PLA, non-energetic placebo; FS, Feeling Scale; FAS, Felt Arousal Scale; AD ACL, Activation–Deactivation Adjective Check List.

The present study was part of a randomized, double-blinded, counterbalanced trial described in detail elsewhere [39]. Briefly, the presented study included 1 baseline visit and 5 experimental trials separated by 48–72 h (Figure 1). During laboratory visit 1, participants had their height and weight measured using a stadiometer and balance-beam scale (Detecto^®^, Webb City, MO, USA), respectively, and their body composition was assessed via bioelectrical impedance analysis (BIA 450; Biodynamics Corp, Shoreline, WA, USA). Participants also had their muscular strength assessed via one-repetition maximum (1-RM) tests performed on the following exercise machines: chest press, leg press, lat pull-down, shoulder press, leg extension and leg curl (Precor^®^, Woodinville, WA, USA). Lastly, participants were also familiarized with the metabolic testing reported in our previous study [39] and were randomly assigned to consume 1 of 5 pre-sleep supplements the night before their first experimental trial. Prior to each experimental trial, participants were asked to refrain from alcohol, caffeine and exercise for 24 h. Participants reported to the laboratory for each experimental trial the morning after consuming their assigned pre-sleep supplement, upon waking and in a fasted state between 06:00 and 09:00. Assessments of appetite, affect and salivary cortisol were conducted before and after the RE bout during the 5 experimental trials (visits 2–6).

### 2.3. Pre-sleep Protein Treatments

Upon completion of baseline testing, participants were randomly assigned to consume 1 of 5 pre-sleep supplements the night before each experimental trial: (1) 24 g CP (34 g, 120 kcals, 24 g protein, 4 g carbohydrate, 1 g fat(; (2) 48 g CP (68 g, 240 kcals, 48 g protein, 8 g carbohydrate, 2 g fat); (3) 24 g WP (31 g, 120 kcals, 24 g protein, 4 g carbohydrate, 1 g fat); (4) 48 g WP (62 g, 240 kcals, 48 g protein, 8 g carbohydrate, 2 g fat); (5) PLA: Propel Zero^TM^, (The Gatorade Company, Chicago, IL, USA; 2.9 g, 0 kcals (placebo)). The protein supplements (Optimum Nutrition, Aurora, IL, USA) were in powder form and were matched for flavor (vanilla) and texture, however the PLA differed in flavor and texture. For the present study, a single and double serving of the majority of commercially available protein supplements (24–25 g per serving) were used as our pre-sleep protein treatments. Supplements were provided in a Ziplock^®^ plastic bag (S. C. Johnson & Son, Racine, WI, USA) and participants were instructed to consume each supplement with 12 oz of water at least two hours after dinner and within 30 min before sleep. 

### 2.4. Experimental Trials (Visits 2–6)

#### Resistance Exercise Sessions

Participants performed RE at 60% 1-RM for 2 sets of 10 repetitions with a 3rd set completed to muscular failure on the chest press, leg press, lat pull-down, shoulder press, leg extension and leg curl exercise machines. Participants performed each repetition at a metronome cadence of 30 beats per minute to standardize time under tension and minimize the variation in repetition speed. This equated to a 2-second concentric and 2-second eccentric phase for each repetition. 

### 2.5. Assessment of Appetite

Appetite (hunger, fullness, and desire to eat) were assessed during each experimental trial using visual analogue scale (VAS; [40]). The VAS is a 100-mm horizontal scale anchored at opposing extremes (“not at all” to “extremely”) for each appetite sensation. Participants were asked to rate their feelings for each sensation by placing a vertical line along the 100 mm scale at three time points (1) immediately upon arrival (0 min), (2) immediate after metabolic testing (~30 min, Pre-RE), and (3) immediately after the RE bout (~90 min, Post RE). Higher scores indicated greater feelings for each sensation.

### 2.6. Assessment of Affect

Affect was measured immediately upon arrival (0 min) and after the RE bout (~90 min, Post RE). The circumplex model of affect [41] was assessed using the Feeling Scale (FS; [42]) and the Felt Arousal Scale (FAS; [43]). The FS is used to assess affective valence and employs an 11-point, single-item, bipolar measure of pleasure–displeasure that has a scale ranging from −5 (“Very Bad”) to +5 (“Very Good”). The FAS is used to assess how aroused or activated an individual feels. The FAS uses a six-point, single item measure with anchors at 1 (Low Arousal) and 6 (High Arousal). 

Participants also completed the Activation–Deactivation Adjective Check List (AD ACL) at the beginning and end of each testing session [44,45]. The AD ACL is a 20-item instrument listing various adjectives, and participants are asked to rate how those adjectives describe them at that moment on a scale of 1 to 4 (i.e., “not at all” to “very much so”). The AD ACL assesses the bipolar dimensions of Energetic Arousal (EA) and Tense Arousal (TA), where the EA dimension ranges from Tiredness to Energy and the TA dimension ranges from Calmness to Tension.

### 2.7. Salivary Sampling and Analysis

Salivary samples were collected using the passive drool technique immediately upon arrival to the laboratory and immediately Post-RE performance bout (within 5 min) to measure morning cortisol levels. Salivary cortisol was analyzed via commercially available ELISA kits according to the manufacturer’s instructions (R&D Systems Inc., Minneapolis, MN, USA) at the conclusion of the study. All assays were run in duplicate and intra-assay and inter-assay coefficients of variation (CV) for cortisol were 29% and 8.3%, respectively. Only samples with CV less than 20% were analyzed.

### 2.8. Statistical Analysis:

All statistical analyses were conducted using IBM SPSS, Version 23.0. Significance was set a priori at an alpha level of ≤0.05. A 5 × 3 (treatment by time) repeated-measures analysis of variance (ANOVA) was conducted to measure differences in hunger, fullness and desire to eat during each trial. A 2 × 2 (treatment by time) repeated measures ANOVA was conducted to determine if there were differences in affect across time and between conditions for appetite, affect and cortisol. Correlational analyses were also conducted to determine if there were significant relationships between appetite, affect and cortisol. Data are reported as mean ± standard deviations in tables and mean ± standard errors in figures. 

## 3. Results

### 3.1. Assessment of Appetite

**Figure 2:**
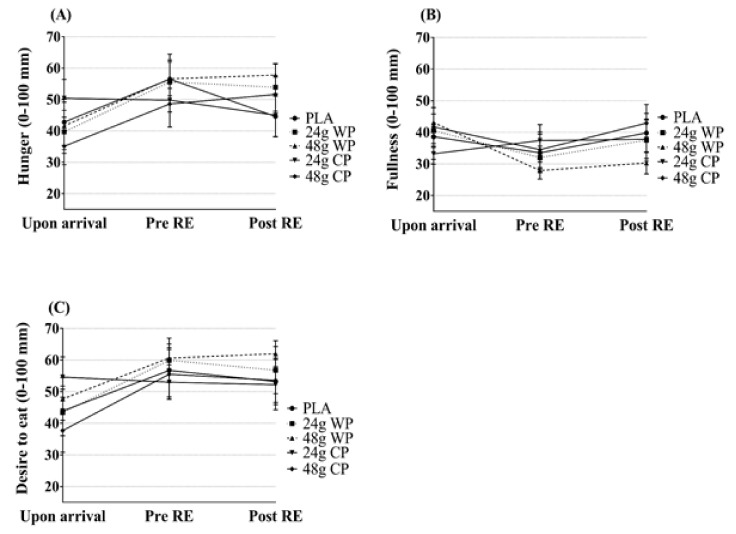
Subjective assessments of appetite (hunger, fullness and desire to eat; mean ± SEM): (**A**) hunger; (**B**) fullness; and (**C**) desire to eat. PLA = placebo; RE = Resistance Exercise; WP = Whey Protein; CP = Casein Protein.

The subjective assessments of hunger, satiety and desire to eat are presented in Figure 2. There were no significant condition × time interactions for hunger, satiety and desire to eat (F (24, 216) = 1.331, *p* = 0.15). Furthermore, there were no significant main effects of condition (F (12, 108) = 0.249, *p* = 0.99) and time for any measures of appetite (F (6, 34) = 1.103, *p* = 0.38).

### 3.2. Affect

**Figure 3:**
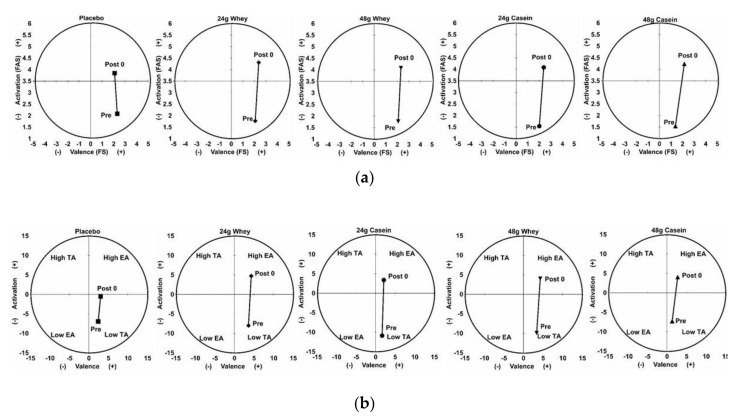
(**a**) Combined group Feeling Scale (FS) and Felt Arousal Scale (FAS) and (**b**) AD ACL responses immediately upon arrival (0 min; Pre-RE) and immediately after resistance exercise performance bout (~90 min; Post-RE) the next morning after pre-sleep protein consumption; n = 14, EA, energetic arousal; TA, tense arousal.

The affective responses to RE are displayed in Table 2 as well as Figure 3. For FS and FAS, there was a significant main effect for time (F (2, 11) = 48.56, *p* < 0.001), but not for condition (F (5, 8) = 0.29, *p* = 0.94) or condition × time interaction effect (F (5, 8) = 0.54, *p* = 0.79). Examination of the univariate analyses shows that the time effect was due only to changes in FAS (F (1, 12) = 62.6, *p* < 0.001). FAS was found to significantly increase following resistance exercise (see Figure 3a). Similarly, for the AD ACL, there was also a significant main effect for time (F (2, 8) = 13.88, *p* = 0.003), but not for condition (F (8, 2) = 1.97, *p* = 0.38) or condition × time interaction effect (F (8, 2) = 0.35, *p* = 0.89) for EA and TA. Univariate analyses found that condition effect was due to changes in both EA (F (4, 36) = 19.64, *p* = 0.002) and TA (F (4, 36) = 30.70, *p* < 0.001) with both showing increases following exercise (see Figure 3b). Based on the circumplex model of affect, these changes suggest that the primary changes in affect were an increase in activation or arousal with little change in valence, similar to what was seen with FS and FAS. 

### 3.3. Cortisol Responses

**Figure 4:**
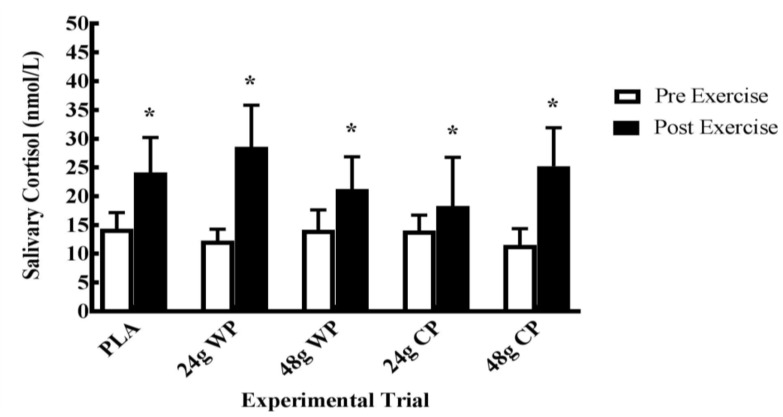
Salivary cortisol concentration changes to resistance exercise performance bout (Mean ± SEM). PLA = placebo; WP = Whey Protein; CP = Casein Protein. *, significantly different from Pre-RE (*p* < 0.05).

Figure 4 presents the salivary cortisol samples that were analyzed. Three samples had a CV greater than 20% and were excluded from analyses. There were no significant condition × time and condition effects for salivary cortisol. However, there was a significant main effect of time observed with cortisol levels increasing from immediately upon arrival to immediately after the RE performance bout (F (1, 10) = 11.3, *p* = 0.007) (See Figure 4).

### 3.4. Relationship between Appetite, Affect and Cortisol

Due to there being no condition or condition × time interactions for any of the dependent variables of interest, conditions were collapsed to perform correlational analyses between the dependent variables. All correlations can be seen in Table 3. Pre-RE, TA was positively correlated with hunger (r = 0.25, *p* = 0.047) and desire to eat (r = 0.35, *p* = 0.005). Cortisol was not related to either affect or appetite pre-RE. Post-RE, there were no significant relationships between appetite and either affect or cortisol. However, cortisol was found to be significantly related to FS (r = 0.32, *p* = 0.018), FAS (r = 0.33, *p* = 0.015) and EA (r = 0.32, *p* = 0.018). 

## 4. Discussion

The present study is the first to investigate the effect of consuming a CP or WP supplement pre-sleep on next morning appetite, affective and salivary cortisol responses prior to and after an RE bout. Contrary to our hypothesis, consumption of 48 g of CP did not result in a greater feeling of fullness when compared to 24 g CP, WP, and a non-caloric PLA in physically active men and women. Similarly, there were no differences in affect and cortisol responses between the groups.

Our finding of no differences between CP and WP on next morning subjective measures of appetite when consumed pre-sleep are consistent with previous acute pre-sleep studies in physically active men [13] and obese women [14]. Similarly, when compared to a non-caloric PLA, neither CP nor WP elicited a more favorable appetite profile in active men [13] and obese men [46], in agreement with the results of the present study. However, Kinsey et al. [14] reported increased fullness and decreased desire to eat in obese women the morning after pre-sleep consumption of 30 g CP, 30 g WP or 34 g maltodextrin when compared to a morning visit when no calories were consumed at least two hours pre-sleep [14]. Thus, it appears that an acute pre-sleep snack (≤240 kcals) in liquid form has an unclear impact on next morning appetite. 

In addition to the present study, only one other study [15] has investigated appetite ratings surrounding a morning exercise bout after consuming a pre-sleep liquid meal. In competitive female runners, a protein and carbohydrate beverage in the form of chocolate milk (~180 kcal) consumed 30 min pre-sleep resulted in a small decrease in hunger and a small increase in satiety prior to the running time trial when compared to a non-caloric PLA. In contrast, our study did not observe any differences between both the low (24 g; 120 kcals) and high (48 g; 240 kcals) doses of CP and WP before and after an RE bout when compared to PLA. However, the study by Ormsbee et al. [15] only assessed appetite prior to exercise, therefore the role of pre-sleep consumption of food to influence morning appetite before and after exercise needs to be further explored. Such findings may have implications for individuals seeking to exercise in the early morning but do not have the adequate amount of time to consume a meal and allow it to digest prior to exercising [47,48]. Adverse appetite profiles and increased hunger may negatively impact exercise performance as well as the motivation to adhere to exercise. With this in mind, the finding that TA was related to the appetite variables of hunger and desire to eat is particularly important. While TA may not be considered a true measure of mood, this finding is similar to that of Hepworth and colleagues [16], who suggest that negative mood leads to eating as a form of affect regulation [16,49,50]. Additionally, in the context of exercise it is thought that people exercise to regulate affect as well, to either decrease negative affect or increase positive affect [49]. Hsiao and Thayer [51] found that mood-related reasons are common explanations for why people exercised, especially in those who were more active. Interestingly, in this study of experienced exercisers, an improvement in affective valence was not seen following exercise. This is not consistent with what is typically seen following aerobic exercise [21] or RE [24], where affect is typically seen to be more positive. However, the increases in both TA and EA are consistent with Thayer’s theory [45] where he proposed at low to moderate energy expenditure they would be related to one another. This increase in both TA and EA, in this case, results in only an increase in activation following exercise, but not valence. According to Thayer’s theory, if intensity was further increased EA would continue to increase and TA would decrease resulting in a net improvement in affect because the reduction in TA would result in an increase in calmness, and hence a greater positive affect.

As noted previously, there were no changes in appetite. However, cortisol increased post-RE in all conditions, which would be consistent with the belief that it is acting as a stress hormone acting in a catabolic nature to maintain blood glucose levels. Interestingly, cortisol was found to be positively related to FS, FAS and EA following RE. Previous studies have found cortisol to be related to more negative and less positive affect [29,30,31,33], but this is usually in a less active condition or not taking into consideration activity level. Few studies have examined the cortisol and affect relationship in exercise. A study by Rudolph and McAuley [34] found an inverse relationship between FS and cortisol post-run. However, the positive relationship in this study could be due to how the stress of RE was interpreted as a positive by the exercisers in this study. The increases in activation as seen by both the FAS and EA may also be evidence that the stressor and increased arousal was normal. This may not be the case for those who are less active. 

The present study did have a few limitations. First, the sample size was relatively small; this may be due to the time demands of our study (6 sessions and 11 total h), as well as the time of day the study was conducted. However, this sample size is consistent with previous pre-sleep studies which have ranged from 11 to 14 participants [13,15,46,52]. Second, the experimental trials for the female participants were not scheduled around the phases of the menstrual cycle. Therefore, we were not able to control for any hormonal changes between trials. Future studies may need to assess participants overnight to elucidate the extent to which appetite is modulated in the post-prandial period after a pre-sleep meal. It is likely the time period between the pre-sleep protein consumption and laboratory arrival (7–9 h) was too long to detect any differences. In addition, considering the differences in total body mass and body composition that exists among participants, future studies should seek to determine the effect of pre-sleep protein prescribed at a dose relative to body mass on appetite and affective responses. 

## 5. Conclusions

The present study demonstrated that pre-sleep consumption of 24 g or 48 g of either CP and WP does not affect next morning appetite, affect and cortisol levels. It is important to note that pre-sleep consumption of protein did not negatively impact next-morning appetite (increase hunger, decrease fullness) when compared to PLA. However, TA was related to increased hunger and desire to eat, suggesting that affect may play an important role in what people may choose to do in the morning, possibly eat or exercise. Further investigation needs to continue to examine the role of RE on affective response, as affect is hypothesized to influence adherence to exercise.

## Figures and Tables

**Table 1:** Descriptive statistics for participants by gender.

Variable	Men (n = 5)	Women (n = 9)
Age	21.6 ± 2.0	25.3 ± 6.4
Height (m)*	1.85 ± 0.1	1.64 ± 0.1
Weight (kg)*	84.8 ± 7.9	61.0 ± 4.8
BMI (kg/m^2^)	24.7 ± 2.9	22.6 ± 2.4
Body Fat (%)	17.7 ± 5.7	22.0 ± 6.2
Maximal Strength Measures (1-RM)		
Chest Press (kg) *	118.7 ± 35.2	45.0 ± 10.3
Leg Press (kg) *	162.6 ± 65.6	103.3 ± 20.1
Lat. Pull Down (kg) *	89.1 ± 20.4	46.2 ± 5.3
Leg Extension (kg) *	155.9 ± 0.0	117.3 ± 20.0
Shoulder Press (kg) *	97.6 ± 35.3	34.9 ± 7.2
Leg Flexion (kg) *	137.0 ± 26.6	79.3 ± 18.9

Mean ± SD; BMI = body mass index; 1-RM = one repetition maximum. * Significant difference between the genders (*p* < 0.05).

**Table 2:** Means (± SD) of affective responses to resistance exercise.

Variable	Placebo	24 g Casein	48 g Casein	24 g Whey	48 g Whey
Pre FS	2.3 ± 1.8	2.0 ± 2.0	1.4 ± 1.8	2.1 ± 1.8	2.1 ± 2.3
Post FS	2.1 ± 2.4	2.4 ± 1.0	2.2 ± 2.2	2.4 ± 2.4	2.3 ± 1.8
Pre FAS	2.1 ± 1.3	1.5 ± 0.8	1.5 ± 0.7	1.8 ± 1.1	1.8 ± 1.0
Post FAS	3.9 ± 1.1	4.1 ± 1.0	4.2 ± 0.9	4.3 ± 1.0	4.1 ± 1.1
Pre EA	21.8 ± 6.6	18.5 ± 6.5	20.8 ± 6.7	21.9 ± 6.7	20.4 ± 6.6
Post EA	26.8 ± 5.8	28.9 ± 3.2	29.8 ± 7.1	31.4 ± 4.3	30.9 ± 2.5
Pre TA	18.4 ± 5.2	16.1 ± 4.0	18.9 ± 4.5	16.8 ± 4.7	15.6 ± 3.4
Post TA	22.5 ± 5.6	25.9 ± 4.1	25.9 ± 3.4	25.3 ± 3.8	24.8 ± 3.0

FS, Feeling Scale; FAS, Felt Arousal Scale; EA, energetic arousal; TA, tense arousal.

**Table 3:** Correlations between affect, appetite and cortisol before and after resistance exercise.

Variable	FS	FAS	EA	TA	Hungry	Fullness	Desire
**Before Resistance Exercise**							
FS	–	–	–	–	–	–	–
FAS	0.25 *	–	–	–	–	–	–
EA	0.54 **	0.58 **	–	–	–	–	–
TA	0.18	0.48 **	0.47 **	–	–	–	–
Hungry	0.03	0.08	−0.09	0.25 *	–	–	–
Fullness	0.06	0.03	−0.01	−0.11	−0.49 **	–	–
Desire	0.07	0.11	−0.02	0.35 **	0.89 **	−0.33 **	–
Cortisol	0.13	0.03	0.21	0.08	−0.08	−0.01	−0.15
**After Resistance Exercise**							
FS	–	–	–	–	–	–	–
FAS	0.40 **	–	–	–	–	–	–
EA	0.63 **	0.45 **	–	–	–	–	–
TA	0.25 *	0.39 **	0.59 **	–	–	–	–
Hungry	−0.06	−0.10	−0.14	−0.06	–	–	–
Fullness	−0.10	−0.06	−0.04	0.20	−0.50 **	–	–
Desire	−0.02	−0.08	−0.15	−0.09	0.89 **	−0.50 **	–
Cortisol	0.32 *	0.33 *	0.33 *	0.10	−0.07	−0.23	−0.12

FS, Feeling Scale; FAS, Felt Arousal Scale; EA, energetic arousal; TA, tense arousal. *, correlation is significant at the 0.05 alpha level. **, correlation is significant at the 0.01 alpha level.
